# Differential Mechanisms of Activation of the Ang Peptide Receptors AT1, AT2, and MAS: Using *In Silico* Techniques to Differentiate the Three Receptors

**DOI:** 10.1371/journal.pone.0065307

**Published:** 2013-06-03

**Authors:** Jeremy W. Prokop, Robson A. S. Santos, Amy Milsted

**Affiliations:** 1 Department of Biology, Program in Integrated Bioscience, The University of Akron, Akron, Ohio, United States of America; 2 Departamento de Fisiologia e Biofísica, Instituto de Ciências Biológicas, Federal University of Minas Gerais, Belo Horizonte, Minas Gerais, Brazil; Max-Delbrück Center for Molecular Medicine (MDC), Germany

## Abstract

The renin-angiotensin system is involved in multiple conditions ranging from cardiovascular disorders to cancer. Components of the pathway, including ACE, renin and angiotensin receptors are targets for disease treatment. This study addresses three receptors of the pathway: AT1, AT2, and MAS and how the receptors are similar and differ in activation by angiotensin peptides. Combining biochemical and amino acid variation data with multiple species sequence alignments, structural models, and docking site predictions allows for visualization of how angiotensin peptides may bind and activate the receptors; allowing identification of conserved and variant mechanisms in the receptors. MAS differs from AT1 favoring Ang-(1–7) and not Ang II binding, while AT2 recently has been suggested to preferentially bind Ang III. A new model of Ang peptide binding to AT1 and AT2 is proposed that correlates data from site directed mutagenesis and photolabled experiments that were previously considered conflicting. Ang II binds AT1 and AT2 through a conserved initial binding mode involving amino acids 111 (consensus 325) of AT1 (Asn) interacting with Tyr (4) of Ang II and 199 and 256 (consensus 512 and 621, a Lys and His respectively) interacting with Phe (8) of Ang II. In MAS these sites are not conserved, leading to differential binding and activation by Ang-(1–7). In both AT1 and AT2, the Ang II peptide may internalize through Phe (8) of Ang II propagating through the receptors’ conserved aromatic amino acids to the final photolabled positioning relative to either AT1 (amino acid 294, Asn, consensus 725) or AT2 (138, Leu, consensus 336). Understanding receptor activation provides valuable information for drug design and identification of other receptors that can potentially bind Ang peptides.

## Introduction

**Figure 1:**
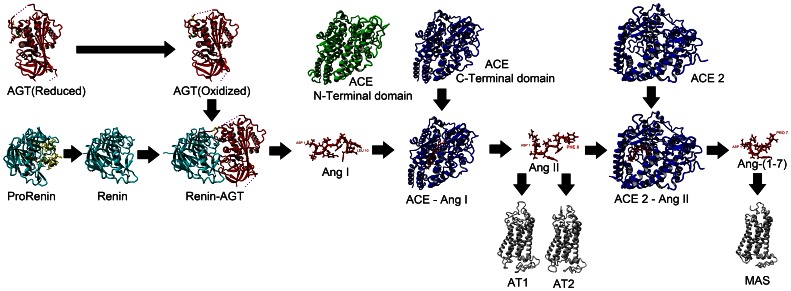
The renin-angiotensin system shown in protein structures based on available or modeled structures. Angiotensinogen (AGT, red) is cleaved by Renin (cyan) producing the ten amino acid Ang I peptide. Ang I is then cleaved by ACE to produce Ang II that is subsequently cleaved by ACE 2 to produce Ang-(1–7). These peptides then bind to AT1, AT2, or MAS (gray).

The renin-angiotensin system (RAS) is a critical homeostatic pathway controlling blood volume and pressure. The pathway is central to homeostasis of blood pressure, and perturbation of steps in this pathway is associated with disease phenotypes, including hypertension, cardiac hypertrophy and fibrosis (reviewed in [1]). In addition, products or components of the RAS influence many other physiological systems such as brain development and reproduction, which is why understanding the details of how the RAS functions is of high importance. Structures of many components of the RAS are known (Table 1) or can be modeled, allowing for a protein structural diagram of the RAS (Figure 1). The RAS begins with the expression of angiotensinogen (AGT), which can exist in either a reduced or oxidized state [2]. The enzyme renin is expressed in a non-enzymatic pro-form [3], activated through either binding to the (pro)renin receptor [4] or enzymatic cleavage of the pro-domain. When activated, renin cleaves a ten amino acid peptide from AGT known as Ang I. This peptide is cleaved in various ways resulting in numerous peptides. The most well defined of these peptides is the cleavage of amino acids nine and ten from Ang I resulting in Ang II by enzymes such as ACE. This peptide is then further processed by enzymes such as ACE 2 to yield Ang-(1–7) [5] or by aminopeptidases to yield Ang III (amino acids 2–8 of Ang II) [6]. Having protein structures of each one of these steps allows for critical understanding of details in how each step works, allowing for novel drug design targeted to the critical steps of the pathway.

**Table 1:** Known structures of the renin-angiotensin system.

Protein	pdb ID	Information	Species
**(Pro)renin**	3vcm	Human Prorenin	Human
**Renin**	1bbs	Native	Human
**Renin**	2ren	Native	Human
**Renin**	2v0z	Aliskiren bound	Human
**(Pro)renin receptor**	3lbs	(pro)renin Receptor MBP fusion	Human
**Agt**	2wxw	Oxidized	Human
**Agt**	2wxx	Oxidized	Mouse
**Agt**	2wxy	Reduced	Mouse
**Renin-Agt**	2x0b	Complexed together	Human
**Ang I**	1n9u	Solution structure	Multiple
**ACE N-term**	2c6f	Native	Human
**ACE N-term**	2c6n	Lisinopril bound	Human
**ACE C-term**	1o8a	Native	Human
**ACE C-term**	1o86	Lisinopril bound	Human
**Ang II**	1n9v	Solution structure	Multiple
**ACE2**	1r42	Native	Human
**ACE2**	1r4l	MLN-4760 bound	Human
**Ang-(1–7)**	2jp8	Solution structure	Multiple

The Ang peptides with the most potent effect on the cardiovascular system are Ang II and Ang-(1–7). Ang II is the most studied, with known interactions with AT1 [7] and AT2 [8] receptors. Ang II binds to AT1 eliciting a blood pressure increase [9]/proangiogenic/proliferative effect [10], or to AT2, yielding a blood pressure decrease [11]/antiangiogenic/antiproliferative effect [12] effect. Gene knockout studies of AT2 show increased blood pressure [11], yet animal research with agonists of AT2 has not shown significant lowering of blood pressure, suggesting that AT2 probably serves more of a role in vascular remodeling or inhibition of AT1 (reviewed in [8]). AT1 and AT2 are members of a large family of G-protein coupled receptors (GPCRs), all sharing seven transmembrane helixes. Ang-(1–7) has been shown to activate the proto-oncogene MAS product, stimulating similar pathways as AT2 activated by Ang II [13], [14]. Several highly homologous MAS-related genes have also been suggested to be activated by Ang peptides [15]. Like AT1 and AT2, MAS and its related proteins are GPCRs, all of which fall into class A or Rhodopsin-like GPCRs. As of now, we do not have structures for AT1, AT2, or MAS receptors. The structure of rhodopsin has been used in many studies modeling AT1[16]–[19] and AT2 [20], but less work has been done on modeling MAS. Using these models, it may be possible to determine how the Ang peptides bind to each receptor and how binding alters the structure to active intracellular pathways. GPCRs readily form homo- or heterodimers with other proteins [21], [22], and this likely functions into the intracellular activation of the pathway. Using protein modeling techniques, sequence alignments, molecular dynamics, docking predictions of Ang peptides and known functional data of AT1, AT2, and MAS, it is possible to address both the role of any conserved binding regions for the Ang peptides in these receptors and potential protein-protein interactions with other membrane proteins.

## Materials and Methods

### Generation of Models for AT1, AT2 and MAS

**Figure 2:**
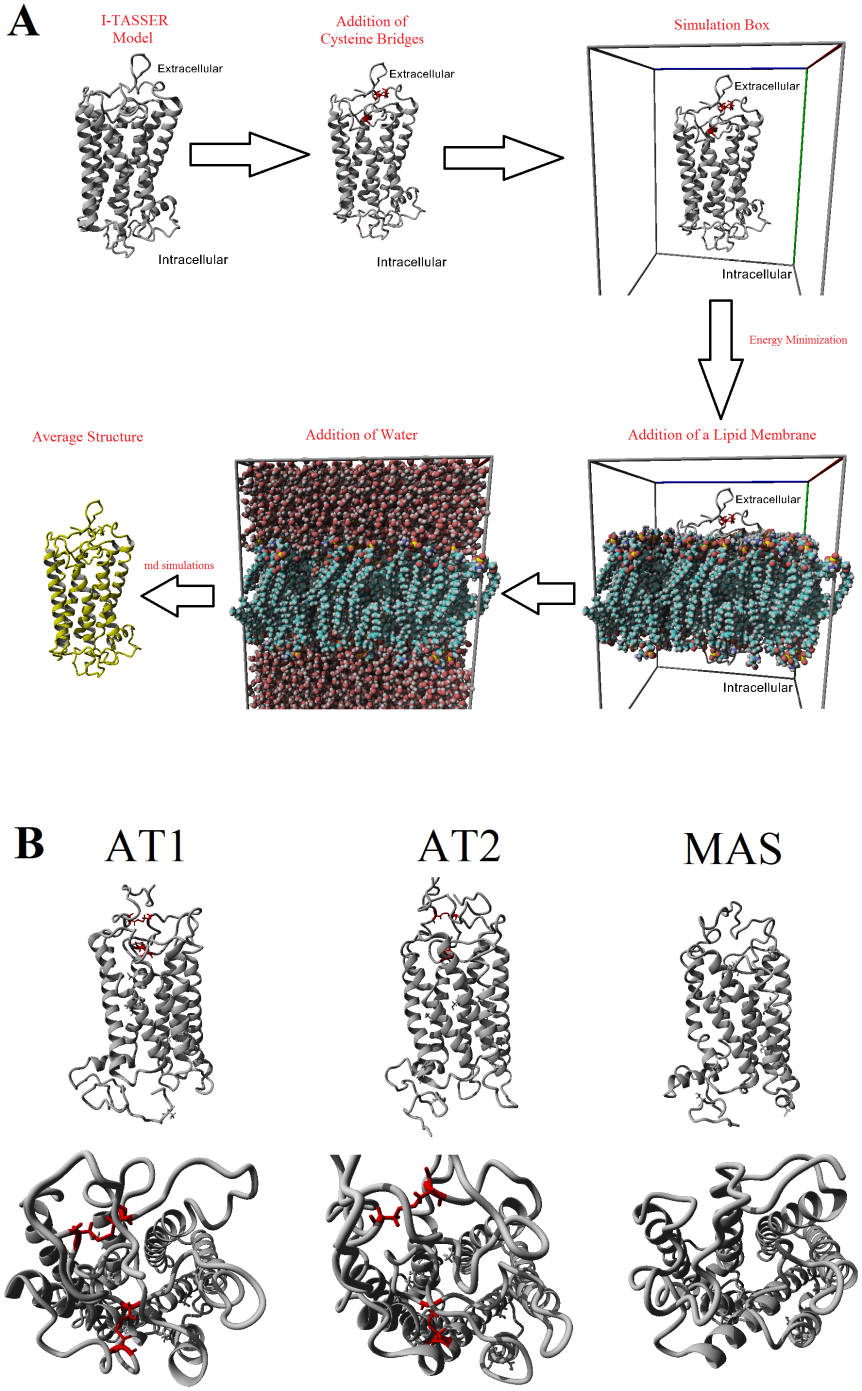
Models for AT1, AT2, and MAS. A) Methodology for creating models of AT1, AT2 and MAS beginning with I-TASSER models, adding cysteine bridges, inserting into a lipid membrane and then running molecular dynamics simulations. B) The averaged models from A for AT1, AT2, and MAS.

Figure 2A shows the methods used to model each receptor. Models for human AT1 [Uniprot: P30556], AT2 [Uniprot: P50052] and MAS [Uniprot: P04201] were created with I-TASSER [23], [24]. Disulfide bonds were added to AT1 and AT2 and energy minimized with AMBER03 [25] force field in 0.997 g/mL of water. The structure of AT1 was then placed into a lipid membrane of phosphatidylethanolamine and simulation run with the standard md_runmembrane macro (http://www.yasara.org/macros.htm) on YASARA. Simulations were run for 2000 picoseconds (ps) of which the first 250 ps were restrained equilibration simulation. The average structure throughout the simulation was used as the model for AT1. The AT2 and MAS models were independently aligned with the AT1/membrane complex, the AT1 removed and simulations run with the md_runmembrane macro. The average structure for each of these was used as the model for each protein (Figure 2B). Alignments of the protein models were performed with Mustang [26] and compared to the structure of Rhodopsin [PDB: 1 gzm] to show similarity in the family.

### Sequence Alignments

Sequences of MAS from multiple species included human [Uniprot: P04201], mouse [Uniprot: P30554], rat [P12526], common chimpanzee [Predicted Gene ID: 472176], macaque (Predicted Gene ID: 703105), naked mole rat [Uniprot: G5BC59], dog [Predicted Gene ID: 484066], and Chinese hamster [Uniprot: G3HGQ0] were aligned using ClustalW. The same was done for AT1 sequences from human [Uniprot: P30556], rat [Uniprot: P25095 and P29089], mouse [Uniprot: P29754], rabbit [Uniprot: P34976], pig [Uniprot: P30555], common chimpanzee [Uniprot: Q9GLN9, Mongolian gerbil [Uniprot: O35210], guinea pig [Uniprot: Q9WV26], dog [Uniprot: P43240], sheep [Uniprot: O77590, chicken [Uniprot: P79785], cattle [Uniprot: P25104], wild turkey [Uniprot: P33396] and AT2 from human [Uniprot: P50052], mouse [Uniprot: P35374], rat [Uniprot: P35351] and Mongolian gerbil [Uniprot: Q9Z0Z6]. The compiled AT1, AT2, and MAS sequences were aligned with the human sequences of each using ClustalW, and the consensus sequences added into the alignment manually. These sequence alignments were compared to the sequence of human rhodopsin [Uniprot: P08100]. Numbering and identification of conserved GPCR amino acids were based on consensus GPCR numbers with AT1 alignments [18]. The numbering system of GPCRs was used with the hundred place as the helix number followed by the next sequential amino acid (101 is the first amino acid on helix one).

### Docking Ang Peptides

To identify the best docking sites in each model, the dock_runensemble macro (http://www.yasara.org/macros.htm) was used with default twenty docking experiments of the ligand on six possible ensembles of the receptor for AT1 or MAS with Ang II or Ang-(1–7). The simulation square was 30 Å on the x, y, and z axis and placed in the proposed binding site. As the initial model had problems with the extracellular domains filling the active site, the region between helix 4 and 5 was deleted to open up the active site. The top ten docking results of each independent run were then treated with the docking_EM_analysis macro (Docking_EM_analysis S1) calculating the potential energy of the receptor, potential energy of the ligand, binding energy of the ligand and movement of the energy minimized structures from the initial structure. For each receptor/ligand data set (containing ten complexes) rankings for the highest value for each binding energy of the ten members of the experiment were made and the scores compiled with the three lowest values selected for further treatment.

The top three of each energy minimized receptor/ligand complex were then analyzed by showing the amino acids conserved among AT1, AT2, and MAS or by binding the ligand to the other receptors with the Docking_EM_top3 macro (Docking_EM_top3 S1). In short, each of the three possible ligand confirmations of the complexes were energy minimized to AT1, AT2, MAS, or Rhodopsin and the potential energy of the receptor and the binding energy of the ligand was calculated. A forced docking experiment (known as initial docking) was also conducted using the known biochemical data of amino acids 512 (Lys) and 621 (His). To create this model the first of the multiple Ang II peptide models as determined by NMR [27] was manually placed so that the C-terminus of Ang II is interacting with amino acid 512 [28], [29] (Lys) and amino acid 8 (Phe) of Ang II interacting with 621 (His) [30]. Twenty manual dockings (all of which had slightly different orientations of amino acid 8) were performed using energy minimizations of the AT1 model in a lipid membrane, and binding energies were calculated to determine the top three forced dockings. These top three were then run through the Docking_EM_top3 macro and compared to the top binding energy of the docking experiments above. Alternatively, a second set of twenty forced dockings (known as buried docking) were performed using the known photolabled data which places the C-terminus close to consensus amino acid 725 (Asn) [31]. These dockings were analyzed in the same manner as the initial docking.

The multiple states of docking were aligned into the structure of AT1 in the lipid membrane using MUSTANG algorithm [26], energy minimized with AMBER03 force field in water, and molecular dynamics performed for 10 nanoseconds.

## Results

**Figure 3:**
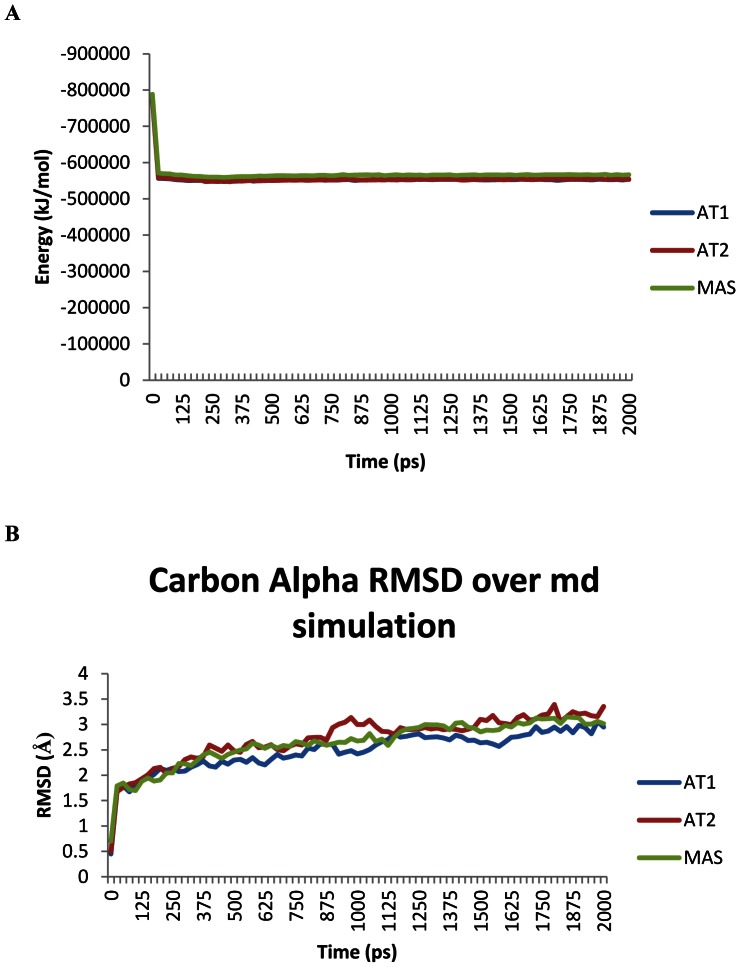
Molecular dynamics of AT1, AT2, and MAS. Simulations of each receptor (AT1, AT2, or MAS) were done in in a lipid membrane for 2 nanoseconds showing either the potential energy of the receptor (**A**) or the averaged carbon alpha root-mean squared deviation (RMSD = average movement of the protein backbone at each amino acid from the initial structure (**B**)).

The Ang peptide receptors AT1, AT2, and MAS are not yet present in the protein data bank (PDB). These structures were modeled (Figure 2A), using a lipid membrane and molecular dynamics simulations to determine the average structure for each (Figure 2B). For all models the structure of Rhodopsin showed low levels of sequence homology (with 14–21% homology) with structures that showed higher homologies (Table 2). The models were threaded primarily using the structure of NK1R [pdb: 2ks9] in I-TASSER, a program highly validated in modeling of GPCRs [32]. Molecular dynamics simulations in the lipid membrane resulted in models of AT1, AT2, and MAS that had similar energies (Figure 3A) and carbon alpha RMSDs (Figure 3B). It should be noted that MAS could potentially contain a Cys bridge, but this bridge was left out of simulations in Figure 3 as it does not significantly alter the energy and movement of the models (Figure S1). Looking at the averaged movement of each amino acid over the entire simulation shows that the seven transmembrane domains are all stabilized in AT1, AT2, and MAS with much higher RMSD in all loops (Figure S2).

**Table 2:** Sequence and structural alignment values of AT1, AT2, MAS, and Rhodopsin.

	Rhodopsin	AT1	AT2	MAS	
**Rhodopsin**	–	19.53%	21.79%	14.51%	
**AT1**	1.862 Å	–	37.80%	20.25%	**% Sequence Homology**
**AT2**	1.963 Å	2.05 Å	–	20%	
**MAS**	2.081 Å	1.938 Å	1.984 Å	–	
		**RMSD of models**			

**Figure 4:**
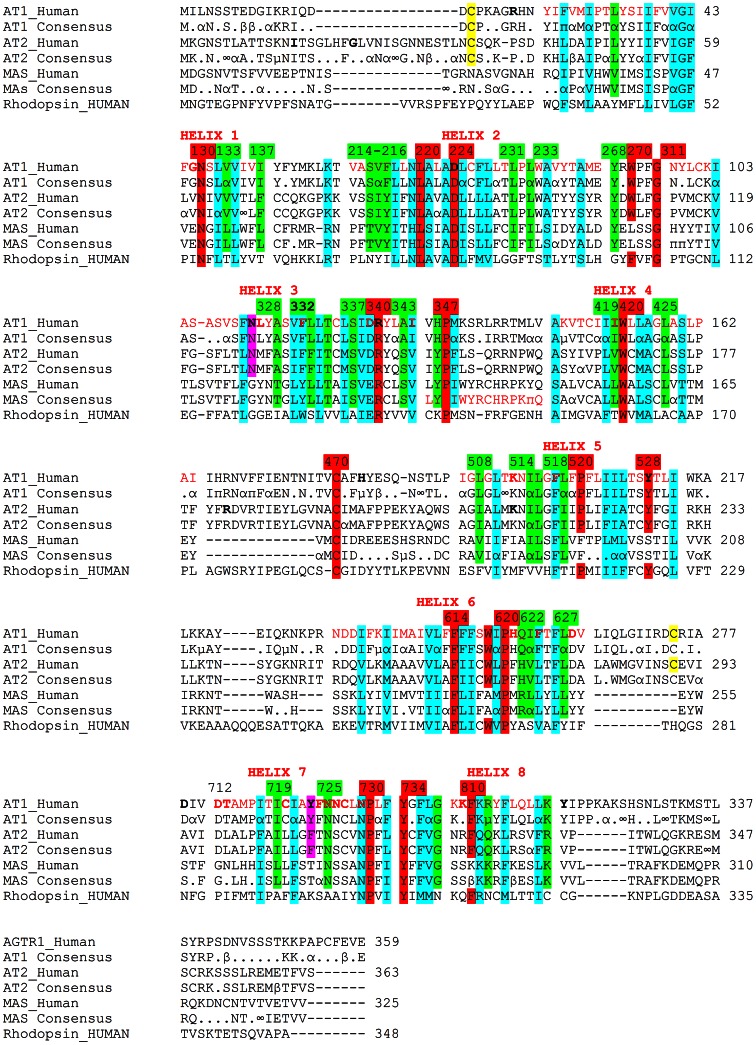
Sequence alignments of AT1, AT2, MAS, and Rhodopsin from human or the consensus sequence. Consensus sequence alignments show those amino acids conserved as a hydrophobic as α (A, V, L, I, F, W, M, P), polar acidic as β (D, E), polar basic as µ (K, R, H), aromatic as π (F, W, H, Y), ∞ for S and T conservation, and. for no conservation. Cysteines highlighted in yellow are those identified to form cysteine bridges, amino acids highlighted in red commonly conserved in GPCR, cyan conserved in all sequences including Rhodopsin, green those conserved only in AT1, AT2, and MAS, and gray/magenta those conserved in only AT1 and AT2 that were identified in other experiments to be critical to Ang peptide binding or activation.

Consensus sequence alignments were used to compare MAS, AT1, and AT2 from multiple species which were then compared to Rhodopsin. AT1 sequences were identified from fourteen species (Figure S3), AT2 from four species (Figure S4), and MAS from eight species (Figure S5). Aligning the human and the consensus sequences from AT1, AT2, and MAS with human Rhodopsin sequences allows for the identification of common GPCR conserved amino acids (Figure 4, red), those conserved in all (Figure 4, cyan), those only conserved in AT1, AT2, and MAS but not Rhodopsin (Figure 4, green) and those conserved in only AT1 and AT2 (Figure 4, gray and magenta). We next identified known natural variants associated with disease as well as mutational data suggesting amino acids of importance (Table S1). The locations of these amino acids were then identified on the sequence alignments (Figure 4, bold and underlined) and conservation in all sequences determined. Of all the natural variants known, only amino acid 517, present as a Phe, is conserved in all three receptors; this is also conserved in Rhodopsin and many other GPCRs. The Table S1 reveals several potentially functional amino acids at 224 (Asp), 336 (Leu), 725 (Asn) and 729 (Asn) that are conserved in all three receptors. Of these only 725 (Asn) is not conserved in Rhodopsin and thus represents a possible target for specific interaction with Ang peptides conserved in AT1, AT2 and MAS.

**Figure 5:**
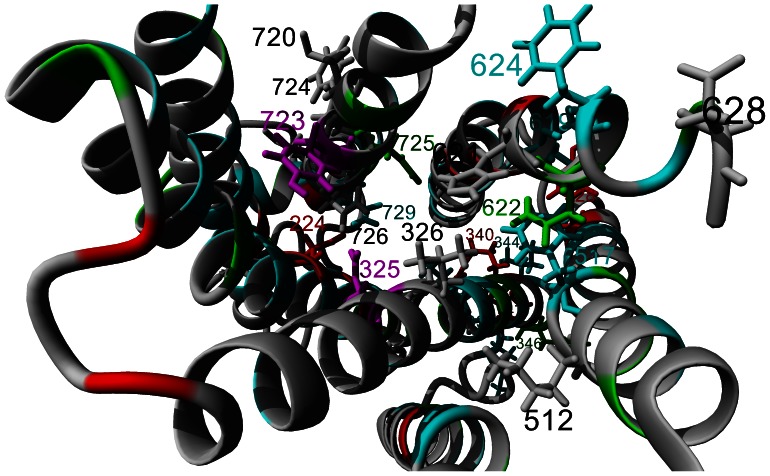
Conservation of amino acids shown on the structure of AT1. View is from looking down the receptor from the extracellular surface**.** Red indicates amino acids commonly conserved in GPCRs, cyan those conserved with Rhodopsin, and green those conserved only in AT1, AT2 and MAS corresponding to Figure 4. Amino acids shown are those identified in Table S1 to have functional roles in Ang peptides binding and activation of receptors, including the consensus GPCR number used.

Combining a structural model of AT1 with the functionally conserved amino acids seen in sequence alignments (using the same coloring for identification of conservation) reveals that amino acid 725 (Asn) is found in the binding pocket of all three receptors (Figure 5). Amino acids 118, 231, 233, 268, 334, 337, 508, 622, and 719 are conserved in the binding pockets of AT1, AT2 and MAS but are not conserved in Rhodopsin (Figure 5, green), all suggesting potential interactions with Ang peptides. Only amino acid 622 (Gln) is currently known to have a functional role in Ang II binding (Table S1). Amino acids in the structure of AT1 (Figure 5) with the amino acid and numbers added, are those that have been identified to contribute to either Ang peptide binding or activation based on previous bench top experiments.

The first very interesting amino acid to have functional importance shown is an Asn at amino acid 325 (Figure 5, magenta). This amino acid when changed in AT1 from an Asn to a Gly leads to an increase activity by the Ang peptide derivative Sar^1^, Ile^4^, Ile^8^ that normally provides no activity [33]. This activation is only seen in inositol phosphate signaling and does not result in phosphorylated receptor [34]. This amino acid is believed to interact with Tyr 4 of Ang II. In MAS, a Gly is found at this site (Figure 4), suggesting that Ang peptides may result in differential activation in MAS. Close by this amino acid is a Tyr (Figure 5, magenta, amino acid 723), which likely interacts with amino acid 325. Tyr 4 of Ang II thus likely displaces this interaction. MAS contains a Thr at amino acid 723, further supporting a differential mechanism of activation by MAS.

**Figure 6:**
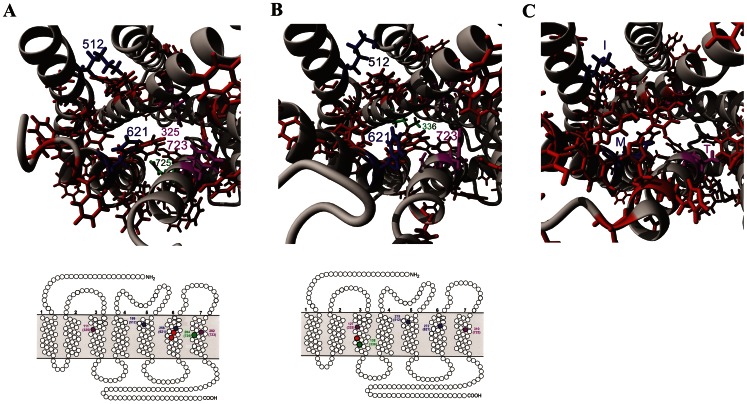
Amino acids involved in activation of AT1 and AT2 but not MAS. Amino acids 512 and 621 (blue) interact with amino acid 8 (Phe) of Ang II, while 325 (magenta) interacts with amino acid 4 (Tyr) of Ang II displacing 723 (Tyr) in both AT1 (**A**) and AT2 (**B**). Aromatic amino acids (red) likely serve to transition Phe 8 from 512 and 621 to the known photolabled interaction sites at 725 for AT1 (**A**) or 336 for AT2 (**B**). The basic seven transmembrane domain schematic representation is added below each figure to show the amino acid positions in both AT1 (**A**) and AT2 (**B**) with the numbers listed at each site the location in the respective protein, and the number in brackets that used as the common numbering scheme. These mechanisms seen in AT1/AT2 are not conserved in MAS (**C**).

Mutational results suggest that amino acid 512 (Lys, amino acid 5.42 in the Ballesteros-Weinstein naming scheme) contacts the C-terminus of Ang II, while amino acid 621 (His, 6.51 in the Ballesteros-Weinstein naming scheme) interacts with amino acid 8 (Phe) of Ang II [30]. Both 512 and 621 are conserved in AT1 and AT2, and both are associated with altered phenotype when mutated (Figure 6A–B, blue). However, MAS has an Ile at amino acid 512 and a Met at 621 (Figure 6C). A second conformation of Ang binding to AT1 based on photolabled experiments shows the C-terminus interacting with an Asn at amino acid 725 [31] (Figure 6A). The structure of AT1, with 512 and 621 identified (Figure 6A, blue), shows aromatic amino acids (Figure 6A, red) that cluster towards amino acid 725. In AT2, however, a Leu at amino acid 336 has been shown to have a photolabled interaction with the C-terminus [35] (Figure 6B, green). In AT2 there is an additional aromatic amino acid (Phe) close to 336 at amino acid 332 that is not found in AT1 (Leu). This is likely the explanation as to why AT1 and AT2 have different photolabled Ang II binding sites. The structure of MAS suggests that the aromatic amino acids would not stabilize the Phe (8) of Ang II (Figure 6C), further suggesting Ang-(1–7) to be the ligand of choice.

Internalization and the pathway of the ligand inside the receptor are more likely to be the main mechanisms of ligand specificity and activation rather than one single binding energy state. Many receptors may contain a site with a high ligand binding rate (static binding), but if the peptides are unable to internalize or unable to transition the receptor into an activated form (dynamic binding), they are biologically inert. AutoDock experiments of both AT1 and MAS for either Ang II or Ang-(1–7), yielded several conformations of high binding energy for the Ang peptides (Figure S6). The top three conformations from each AutoDock experiment were placed onto each of the other receptors and energy minimized (Figure S7). This revealed binding energies for Ang II to be higher on either AT1 or AT2 than that of MAS, while Ang-(1–7) had a similar binding energy to all structures. Visual analysis of the binding of all these experiments shows the Ang peptide to be interacting more extracellular than the mutagenesis data suggests (Figure S8). To combat this, forced docking experiments were performed on AT1 with Ang II’s eighth amino acid Phe interacting with 512/621 (Initial binding) or amino acid 725 (Buried binding). The binding energies for both the internalization (based on AutoDock results above) and the initial binding were lower for MAS than AT1 and AT2, suggesting as to why Ang II has a lower binding affinity for MAS (Figure S9A). However, Ang-(1–7) has similar binding energy for MAS compared to AT1 and AT2 (Figure S9B).

**Figure 7:**
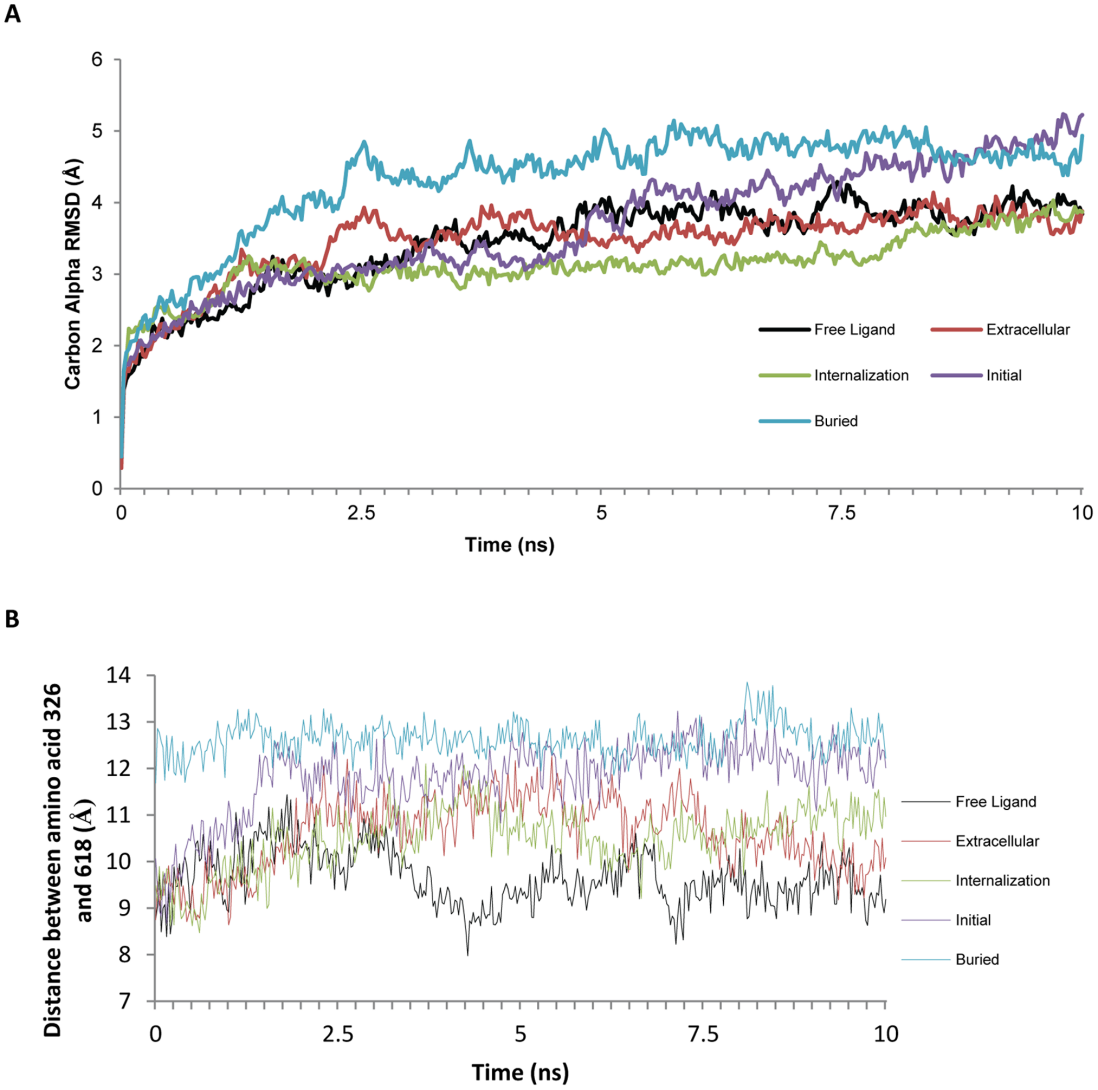
Molecular dynamics simulations of the multiple states of AT1 in activation. The Carbon alpha RMSD of Ang II bound at the multiple points of activation to AT1 (A). The graph shows that the buried position (cyan) yields an increase in overall dynamics of the AT1 receptor. The initial binding (purple) led to a transition in the simulation to yield a similar binding as the buried as the simulation neared 8 ns. **B**) The distance between two of the amino acids (326 and 618) found in the site of AT1 where the eighth amino acid (Phe) comes to the final photolabled interaction for each of the stages of AT1 activation. This shows that the binding of Ang II in the buried position causes a stretching (around 3 Å), leading to opening of new interaction sites for protein interactions. The initial (purple) binding led to propagation and stretching of the receptor around 2 ns yielding similar values as that of the buried binding.

**Figure 8:**
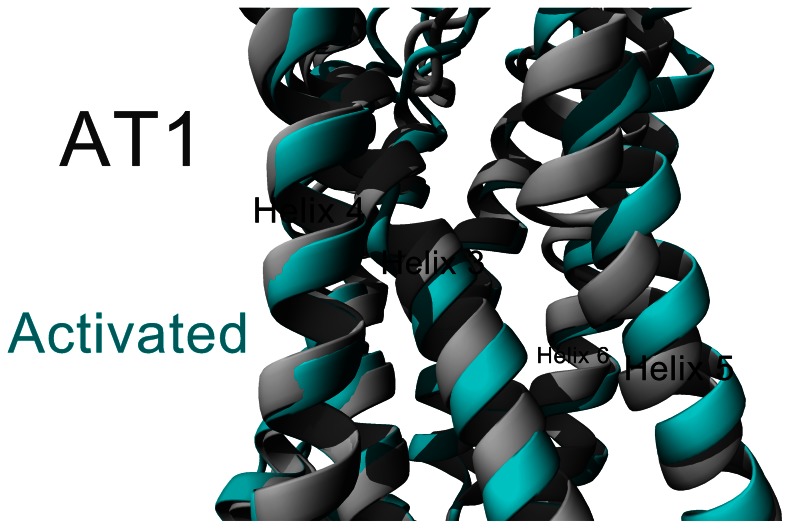
Activated vs. non-activated AT1. The average structure over the 10 ns simulations shown for either AT1 with Ang II free (gray) or in the final buried position (cyan). This shows that Ang II activation likely leads to shifting in helix 3, 5 and 6. This suggests regions of helix 5 (containing the largest movement of the helix) to likely recruit other proteins when Ang II is bound. Additionally some modification made in the intracellular region (due to the shifting of helix 3) could potentially modify intracellular activation.

Molecular dynamics were performed for ten nanoseconds on each of these conformations of AT1. When Ang II was either free from AT1 (Figure 7A, black), interacting with the extracellular loops (Figure 7A, red), or beginning internalization (Figure 7A, green) AT1 had normal carbon alpha RMSDs. When Ang II is bound in the initial binding mode (Figure 7A, magenta) movement started normal, but began increasing around 5 nanoseconds. At this point the movement increased to values seen in the buried binding (Figure 7A, cyan). The stretching of the receptor was observed in the buried binding, and also observed in the initial binding shortly after the simulations started (Figure 7B). The stretching of the receptor can be seen to result in movement of helix 3, a slight rotation of helix 6 and significant movement of helix 5 (Figure 8). Amino acid 8 (Phe) began transitioning towards the buried binding in the initial binding simulation, resulting in the changes starting around 2 nanoseconds.

## Discussion

**Figure 9:**
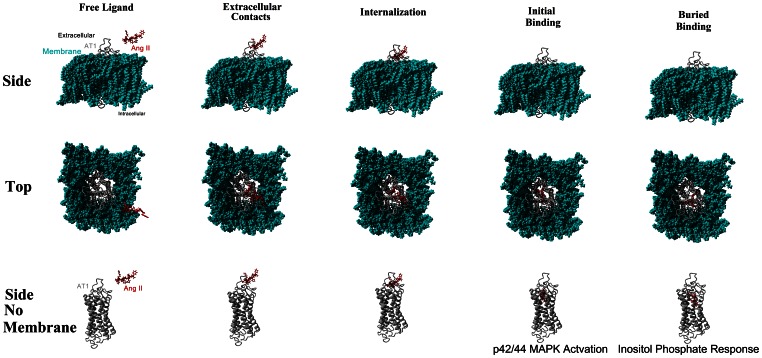
Pathway of activation of AT1 by Ang II. Figure shows the multiple binding states of Ang II activation of AT1 receptor. Initial binding results in movement of helix 7 by Tyr4 of ANG II leading to p42/44 MAPK activation; buried binding results in movement of helix 5 by Phe8 of Ang II leading to Inositol Phosphate response.

Binding and activation of various GPCRs by Ang peptides likely involves multiple binding modes and conformations of the receptor. Therefore, the activation is not static, but involves a very diverse energy landscape for activation. The binding involves extracellular contacts and internalization, which then complex into initial binding and a buried binding mechanism for activation (Figure 9). Many studies in the past have suggested multiple stages of activation; separating receptor phosphorylation, p42/44 MAPK activation, internalization, and inositol phosphate signaling [33], [36]–[40]. In the case of AT1/AT2 we propose that Ang II will first complex into an initial binding with Ang II’s C-terminus bound to amino acid 512 (Lys) of AT1/AT2, and the side chain of the eighth amino acid (Phe) interacting with the aromatic amino acid 621 (His/Phe) of AT1/AT2. At the same time amino acid 4 (Tyr) of Ang II interacts with amino acid 325 (Asn), displacing amino acid 723, leading to rotation of helix seven. This initial binding likely activates the p42/44 MAPK [37]. This binding is confirmed through mutagenic experiments showing that for receptor activation to occur, amino acid 512 needs to be basic [28] and 621 aromatic [30], while Ang II’s eighth amino acid must be aromatic [41].

Photolabeling experiments [31], [35] show the final state of peptide/receptor binding, but in the past these data have been thought to be inconsistent with the mutagenic data. We suggest a hypothesis that includes both data sets as valid, where mutagenic data is consistent with inhibition of the initial binding conformation. The ligand is then internalized from the initial binding mode by passing along conserved aromatic residues (614 and 618) through pi-pi interaction to amino acid 725 (Asn) of AT1. In AT2, the additional aromatic amino acid 332 (Phe) causes the Phe (8) of Ang II to move to 336 (Leu). This buried binding conformation likely induces structural conformation changes to the receptor at helices 3, 5, and 6 resulting in inositol phosphate response. This internalization changes Ang II binding from a horizontal-like conformation (in initial binding) to a vertical conformation (in buried), with the pivot point of Ang II at amino acid four (Tyr). In this change, internal packing of AT1 is disrupted, causing expansion of the middle of AT1 and increased movement of amino acids in this region. This expansion exposes amino acids normally not exposed to the membrane, allowing for recruitment and/or interaction with other membrane proteins. The final position of the Ang binding transition is seen with the photolabled experiments, while all other states of binding require different tools to visualize that binding state, as they are transitions rather than final configurations.

Amino acid 622, which is adjacent to the aromatic residue 621 in the initial binding conformation, is present as a His in AT2. Mutations of this amino acid are known to affect ligand binding [42]. AT2 does not have an aromatic amino acid at 724, which is required for interaction with the C-terminus of Ang II in AT1 [31]. This absence (with the addition of Phe 332) likely leads to a different migration from the initial state to the buried state in AT2. Variation in the final buried site alters the dynamics of a separate region of the GPCR, and thus opens a different possible binding site for recruitment of additional proteins. In our models, we also observed very few amino acids conserved between AT1 and AT2 that would likely interact with the first amino acid of Ang II. Recent evidence suggests that Ang III may be the primary agonist of AT2 in the kidney [43], [44]. Our model suggests that no amino acids have been conserved in the sequence divergence between AT1 and AT2 that would interact with amino acid one of Ang II. Amino acid two (Arg) of Ang II has been shown to interact with amino acid 712 (Asp) of AT1 [45] which is conserved in both AT1 and AT2, but not in MAS.

MAS and its related proteins are not activated by Ang II [15]. Our models and sequence analysis reveal that the internalization process differs in MAS compared to AT1 and AT2. Numerous amino acids are conserved in AT1, AT2, and MAS with amino acids 231 (hydrophobic) and 318 (hydrophobic) contacting Ang II in our initial binding to MAS. With variations at amino acids 512 and 621 (both hydrophobic in MAS), we suggest an unfavorable interaction with Phe (8) of Ang II, which is removed in Ang-(1–7). Amino acid 325 varies in MAS to amino acids that are known to lead to receptor activation (Gly) in AT1 [33], this suggests that Tyr (4) of Ang-(1–7) may insert into this region inducing structural alterations in helix seven. This alteration likely leads to interactions with other membrane proteins. Several other MAS related proteins have been shown to interact with Ang peptides and activate intracellular pathways. Studying the details of these proteins will allow for the predictions of which proteins interact with MAS to yield activation.

Protein activation of AT1 is likely to be through expansion and rotation of the helices. This is further suggested by mechanical stretch of cells transfected with AT1 leading to activation of intracellular signals without agonist induction [46], [47]. These expansions either alter regions on the intracellular side leading to G-protein activation and association with Jak2 through the KYIPP motif [48] or through changing interactions with other membrane bound proteins such as other GPCR [49]. AT1 has been shown to homo-oligomerize [50], or hetero-oligomerize with AT2 [51], Bradykinin B2 receptor [21], [52], MAS [53], and CB_1_R [54].

In addition to Ang-(1–7), many metabolites of Ang peptides are found. This makes addressing the role of the receptors and other associated proteins such as the angiotensin receptor associated protein (ATRAP) difficult using classical physiology and biochemistry approaches. The use of computational tools expands our current research approach to study possible mechanisms existing for these various components. Having detailed molecular/atomic mechanism provided in this study, allows for new analyses of other Ang peptides such as Ang III and Ang IV, addressing whether they have similar mechanism of activation of AT1 and AT2. Having identified specific amino acids of the receptor that lead to activation by Ang peptides, we can also begin searching other GPCR sequences for other proteins that have the potential to bind and be activated by Ang peptides.

## Supporting Information

Figure S1 **Dynamics with a disulfide bridge in MAS.** A). Location of the two Cys amino acids on the numbering system of MAS. B). Conservation of amino acids in Mouse, Rat and Human. C). Molecular dynamics simulation of our model 1 of MAS (blue) compared to the model with a Cyc-Cys bridge (red) revealing minimal change in the energy or averaged carbon alpha RMSD over a 1 nanosecond simulation.
(TIF)

Figure S2 **AT1, AT2 and MAS molecular dynamics simulation data for amino acid carbon alpha RMSDs.** Molecular dynamic simulation results showing similar carbon alpha RMSDs for amino acids in the models of AT1, AT2, and MAS in a lipid membrane. The seven transmembrane domains are numbered all showing stability of movement relative to the loops.
(TIF)

Figure S3 **AT1** **sequence alignments from multiple species.** Consensus alignment show amino acids 100% conserved, those conserved as a hydrophobic amino acid as α (A, V, L, I, F, W, M, P), polar acidic as β (D, E), polar basic as µ (K, R, H), aromatic as π (F, W, H, Y), ∞ for S and T conservation, and. for no conservation.
(TIF)

Figure S4 **AT2** **sequence alignments from multiple species.** Consensus alignment show amino acids 100% conserved, those conserved as a hydrophobic amino acid as α (A, V, L, I, F, W, M, P), polar acidic as β (D, E), polar basic as µ (K, R, H), aromatic as π (F, W, H, Y), ∞ for S and T conservation, and. for no conservation.
(TIF)

Figure S5 **Mas sequence alignments from multiple species.** Consensus alignment show amino acids 100% conserved, those conserved as a hydrophobic amino acid as α (A, V, L, I, F, W, M, P), polar acidic as β (D, E), polar basic as µ (K, R, H), aromatic as π (F, W, H, Y), ∞ for S and T conservation, and. for no conservation.
(TIF)

Figure S6 **Top 10 results from the docking ensemble experiment.** Yellow bars are those dockings that went on to the top3 macro analysis from each group.
(TIF)

Figure S7 **Top three em docking macro results from each of the ten top ligand/receptor docking ensemble runs (figure S6 in yellow) analyzed on AT1 (blue), AT2 (red), MAS (green), or Rhodopsin (purple).**
(TIF)

Figure S8 **Structures of the top 3 results of docking (Figure S7) of either AT1 or MAS to either Ang II or Ang-(1–7).**
(TIF)

Figure S9 Binding energy of Ang II (A) through either an Autodock experiment representing internalization (blue), the initial binding (red) as identified by forced docking using mutagenesis data, or the buried binding (green) based on photolabled data. This shows a lower binding energy for MAS at both the internalization and initial thus suggesting why MAS would bind Ang II with a lower affinity than AT1 or AT2. Binding energy for Ang-(1–7) binding however suggests similar energy for all three receptors (B).
(TIF)

Table S1 Amino acids known to have functional roles in AT1, AT2 or MAS with the consensus amino acid # and amino acid found at that location in AT1, AT2, or MAS. A brief description of each is given and the reference for the published role of that amino acid. Some references can be found in the manuscript with additional references listed in the. The amino acid found in each receptor based on sequence alignments is also listed
(XLSX)

Docking_EM_analysis S1 This Macro energy minimizes (EM) the target *in vacuo*, adds water and EM with AMBER 03 force field, then calculates the PE of the receptor/ligand, the BE of the ligand, and the RMSD of initial structure to final structure.
(MCR)

Docking_EM_top3 S1 This Macro analyses the top three results of the docking_EM_analysis macro and compares them to the structure when complexed and EM to AT1, MAS, AT2, and Rhodopsin.
(MCR)

Additional References S1 Material referenced in Table S1
(DOCX)
