# Supplementary figures and images for: Differential Mechanisms of Activation of the Ang Peptide Receptors AT1, AT2, and MAS: Using In Silico Techniques to Differentiate the Three Receptors
Source: PLoS One. 2013 Jun 3;8(6):e65307. doi: 10.1371/journal.pone.0065307 (PMC3670877; doi:10.1371/journal.pone.0065307)

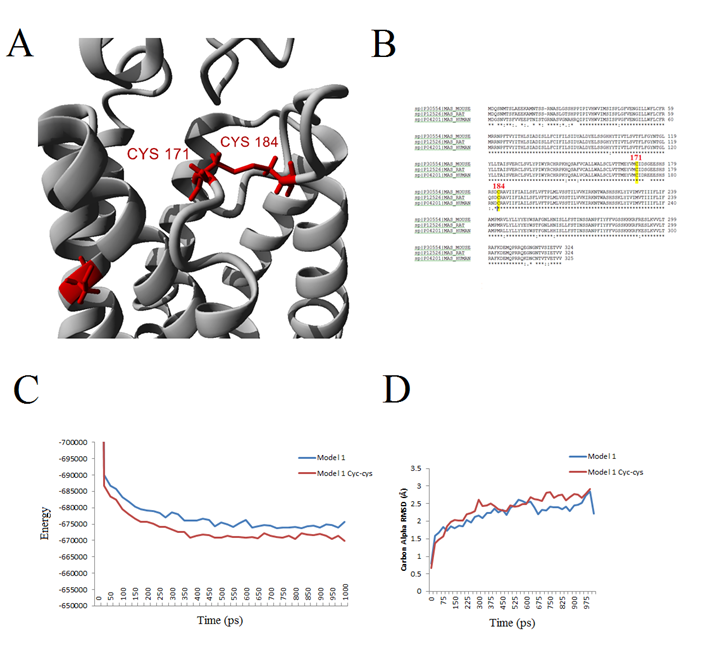

Supplement: Figure S1 — Dynamics with a disulfide bridge in MAS. A). Location of the two Cys amino acids on the numbering system of MAS. B). Conservation of amino acids in Mouse, Rat and Human. C). Molecular dynamics simulation of our model 1 of MAS (blue) compared to the model with a Cyc-Cys bridge (red) revealing minimal change in the energy or averaged carbon alpha RMSD over a 1 nanosecond simulation. (TIF) [file pone.0065307.s001.tif]

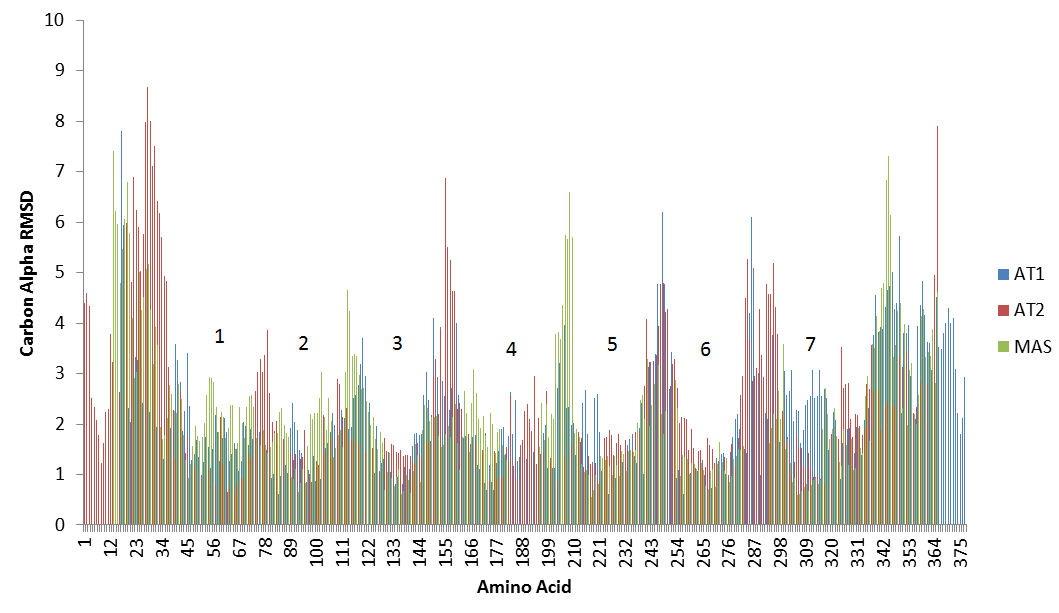

Supplement: Figure S2 — AT1, AT2 and MAS molecular dynamics simulation data for amino acid carbon alpha RMSDs. Molecular dynamic simulation results showing similar carbon alpha RMSDs for amino acids in the models of AT1, AT2, and MAS in a lipid membrane. The seven transmembrane domains are numbered all showing stability of movement relative to the loops. (TIF) [file pone.0065307.s002.tif]

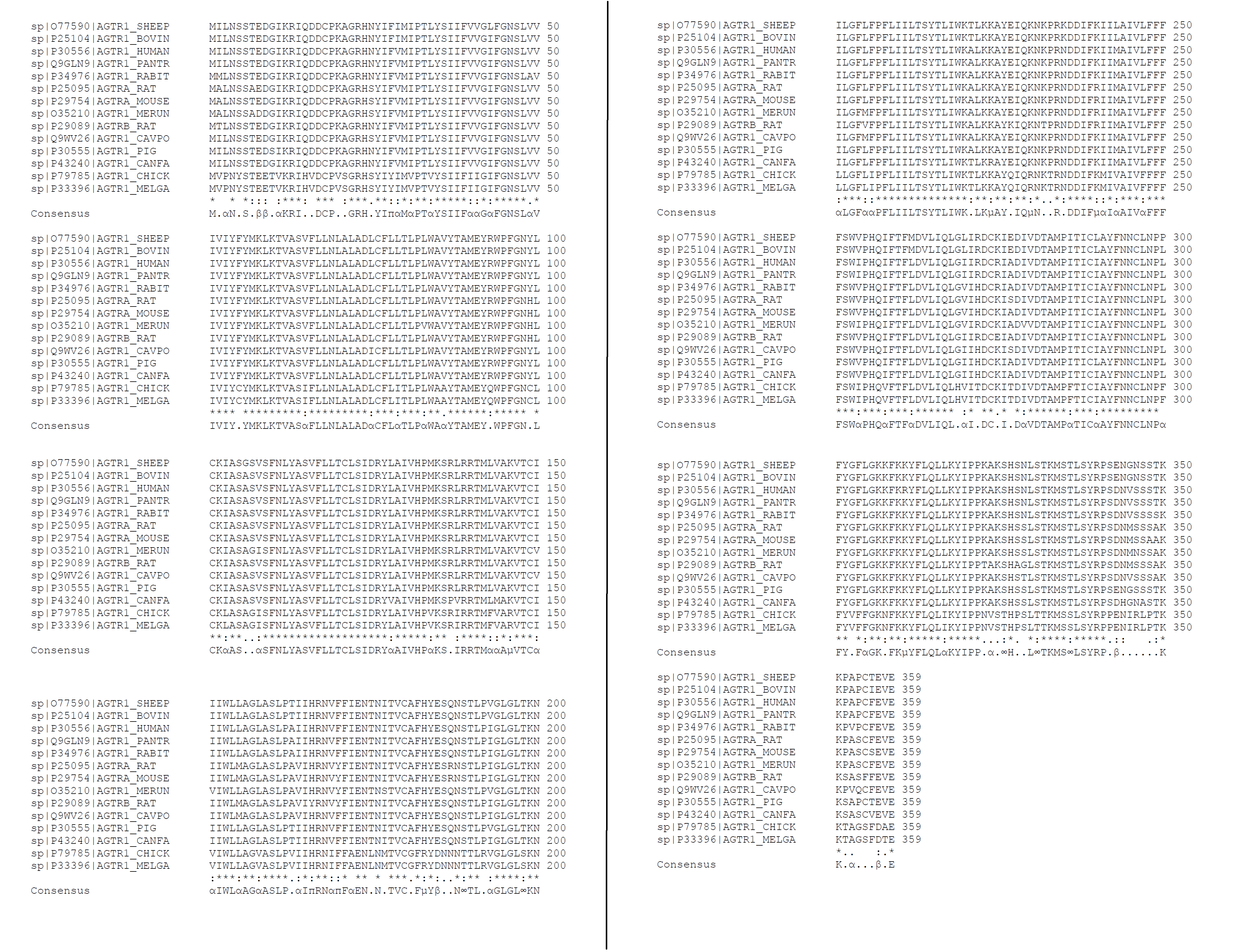

Supplement: Figure S3 — AT1 sequence alignments from multiple species. Consensus alignment show amino acids 100% conserved, those conserved as a hydrophobic amino acid as α (A, V, L, I, F, W, M, P), polar acidic as β (D, E), polar basic as µ (K, R, H), aromatic as π (F, W, H, Y), ∞ for S and T conservation, and. for no conservation. (TIF) [file pone.0065307.s003.tif]

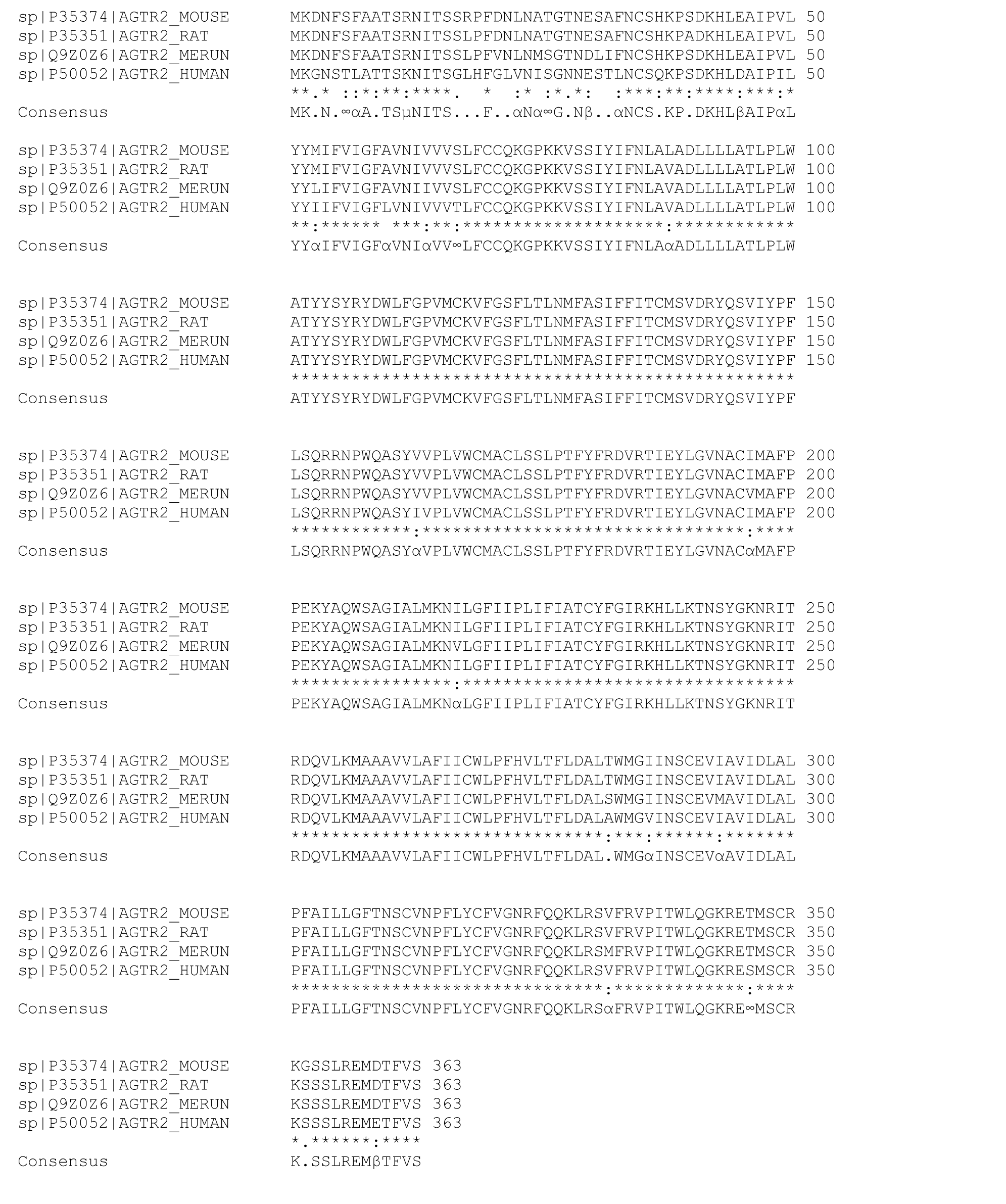

Supplement: Figure S4 — AT2 sequence alignments from multiple species. Consensus alignment show amino acids 100% conserved, those conserved as a hydrophobic amino acid as α (A, V, L, I, F, W, M, P), polar acidic as β (D, E), polar basic as µ (K, R, H), aromatic as π (F, W, H, Y), ∞ for S and T conservation, and. for no conservation. (TIF) [file pone.0065307.s004.tif]

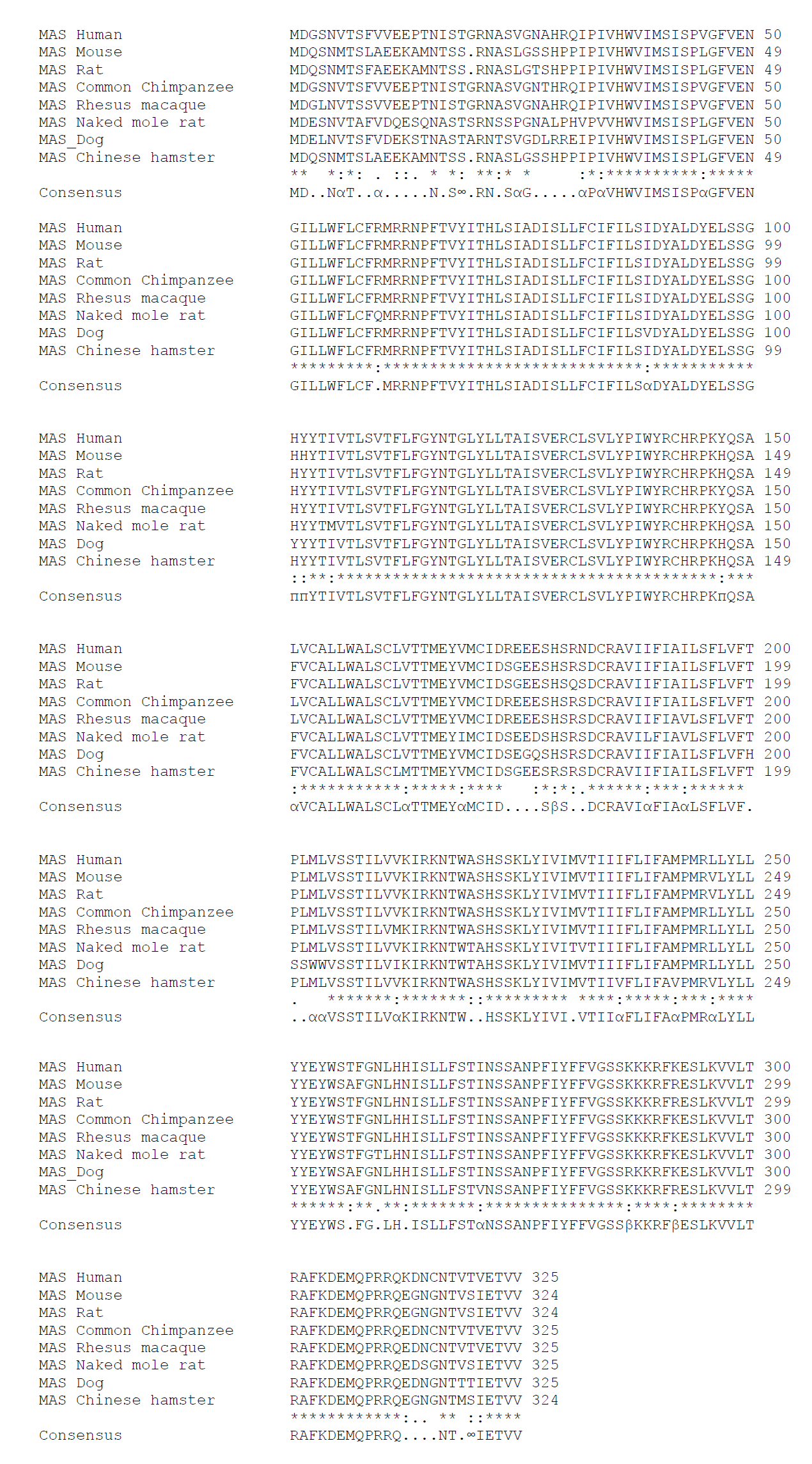

Supplement: Figure S5 — Mas sequence alignments from multiple species. Consensus alignment show amino acids 100% conserved, those conserved as a hydrophobic amino acid as α (A, V, L, I, F, W, M, P), polar acidic as β (D, E), polar basic as µ (K, R, H), aromatic as π (F, W, H, Y), ∞ for S and T conservation, and. for no conservation. (TIF) [file pone.0065307.s005.tif]

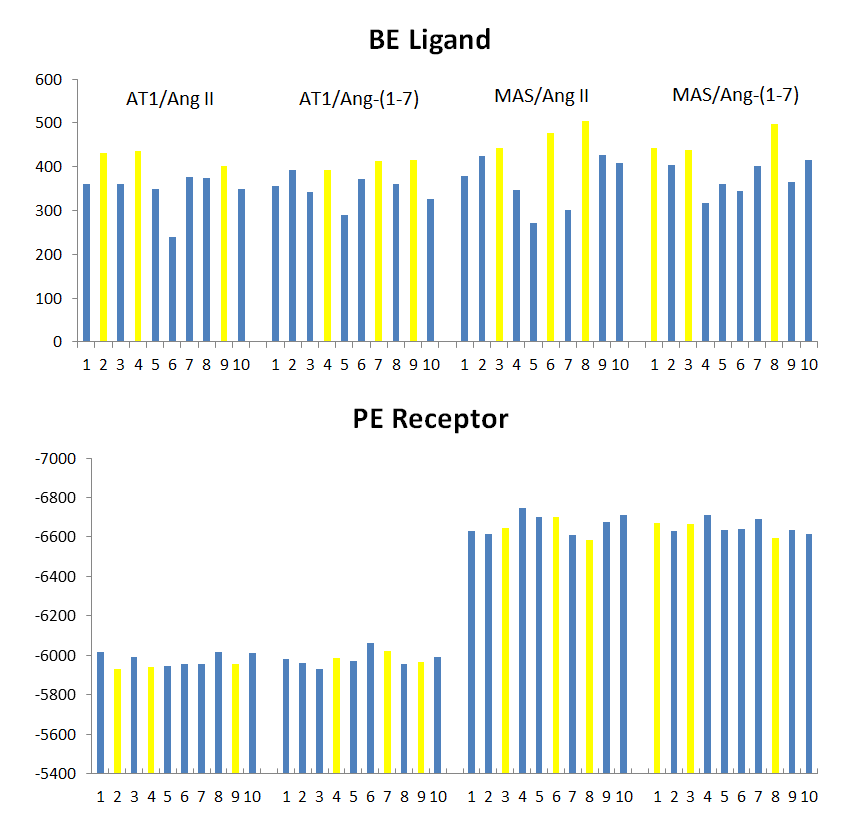

Supplement: Figure S6 — Top 10 results from the docking ensemble experiment. Yellow bars are those dockings that went on to the top3 macro analysis from each group. (TIF) [file pone.0065307.s006.tif]

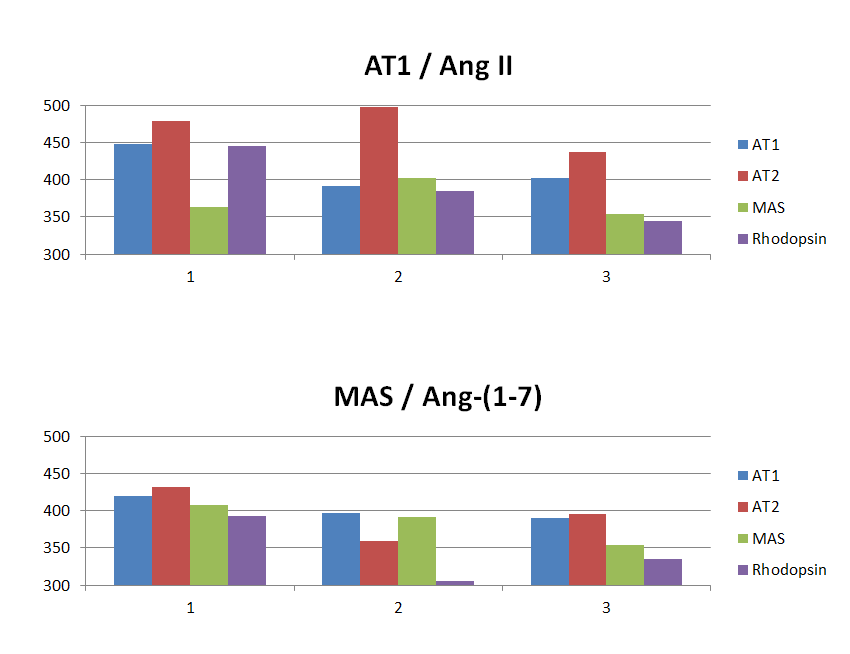

Supplement: Figure S7 — Top three em docking macro results from each of the ten top ligand/receptor docking ensemble runs (figure S6 in yellow) analyzed on AT1 (blue), AT2 (red), MAS (green), or Rhodopsin (purple). (TIF) [file pone.0065307.s007.tif]

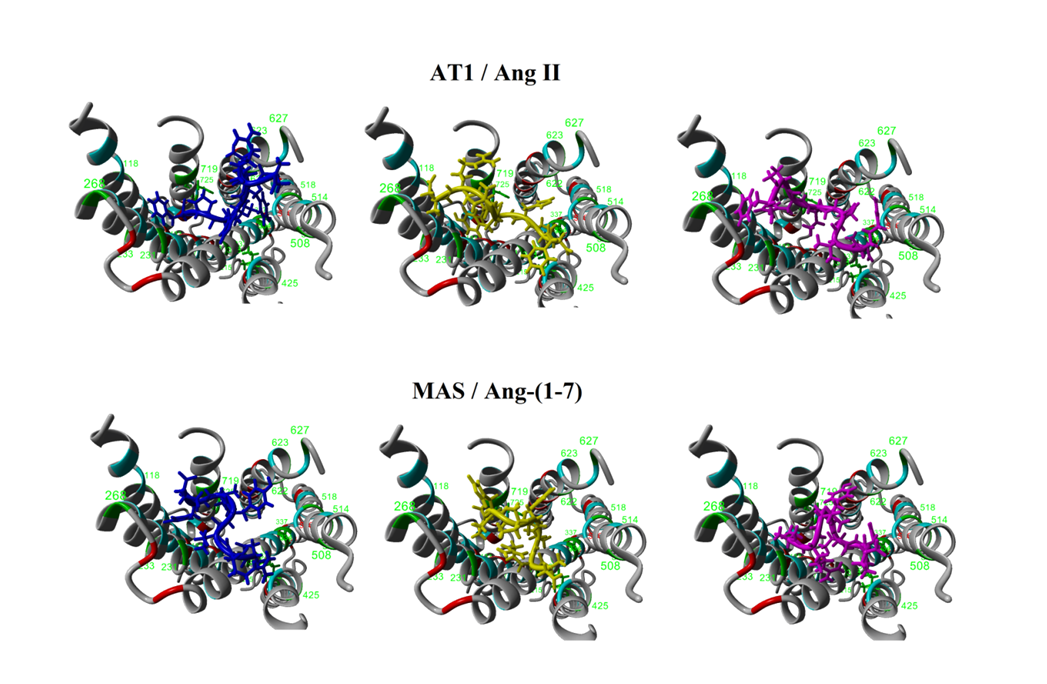

Supplement: Figure S8 — Structures of the top 3 results of docking (Figure S7) of either AT1 or MAS to either Ang II or Ang-(1–7). (TIF) [file pone.0065307.s008.tif]

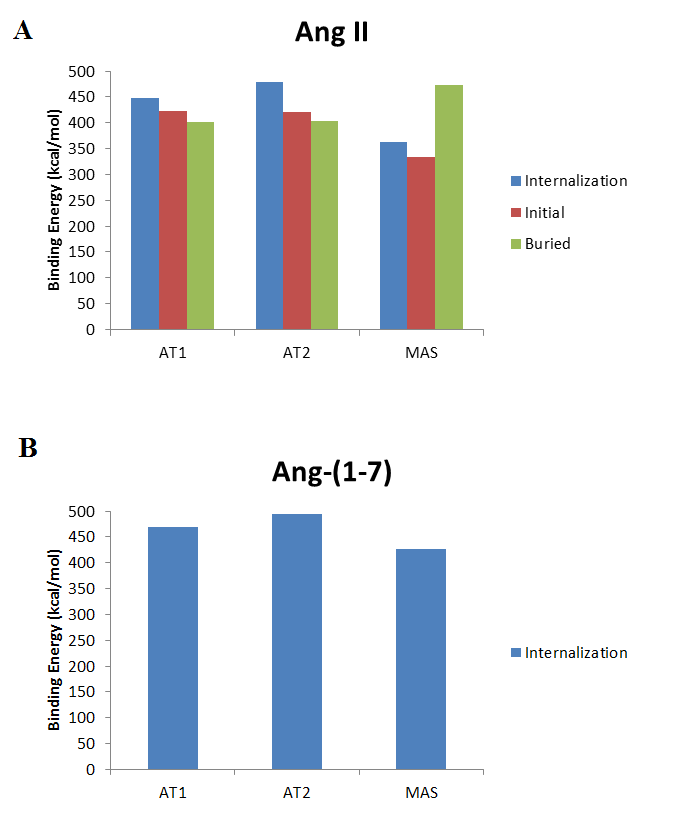

Supplement: Figure S9 — Binding energy of Ang II (A) through either an Autodock experiment representing internalization (blue), the initial binding (red) as identified by forced docking using mutagenesis data, or the buried binding (green) based on photolabled data. This shows a lower binding energy for MAS at both the internalization and initial thus suggesting why MAS would bind Ang II with a lower affinity than AT1 or AT2. Binding energy for Ang-(1–7) binding however suggests similar energy for all three receptors (B). (TIF) [file pone.0065307.s009.tif]
