# Supplementary material for: Differential Mechanisms of Activation of the Ang Peptide Receptors AT1, AT2, and MAS: Using In Silico Techniques to Differentiate the Three Receptors
Source: PLoS One. 2013 Jun 3;8(6):e65307. doi: 10.1371/journal.pone.0065307 (PMC3670877; doi:10.1371/journal.pone.0065307)
Supplement: Additional References S1 — Material referenced in Table S1 (DOCX) [file pone.0065307.s013.docx]

Additional References in Supplemental Table 1

55 Vervoort VS, Beachem MA, Edwards PS, Ladd S, Miller KE, et al. (2002) AGTR2 mutations in X-linked mental retardation. Science 296: 2401–2403.

56 Santos EL, Pesquero JB, Oliveira L, Paiva ACM, Costa-Neto CM (2004) Mutagenesis of the AT1 receptor reveals different binding modes of angiotensin II and [Sar1]-angiotensin II. Regul Pept 119: 183–188.

57 Hansen JL, Haunsø S, Brann MR, Sheikh SP, Weiner DM (2004) Loss-of-function polymorphic variants of the human angiotensin II type 1 receptor. Mol Pharmacol 65: 770–777.

58 Nikiforovich GV, Zhang M, Yang Q, Jagadeesh G, Chen HC, et al. (2006) Interactions between conserved residues in transmembrane helices 2 and 7 during angiotensin AT1 receptor activation. Chem Biol Drug Des 68: 239–249.

# 59 Hunyady L, Zhang M, Jagadeesh G, Bor M, Balla T, et al. (1996) Dependence of agonist activation on a conserved apolar residue in the third intracellular loop of the AT1 angiotensin receptor. Proc Natl Acad Sci USA 93: 10040-10045.

# 60 Gaborik Z, Jagadeesh G, Zhang M, Spat A, Catt KJ, et al. (2003) The role of a conserved region of the second intracellular loop in AT1angiotensin receptor activation and signaling. Endocrinology 144: 2220-2228.

# 61 Heerding JN, Yee DK, Jacobs SL, Fluharty SJ (1997) Mutational analysis of the angiotensin II type 2 receptor: contribution of conserved extracellular amino acids. Regul Pept 72: 97–103.

# 62 Hunyady L, Bor M, Balla T, Catt KJ (1995) Critical role of a conserved intramembrane tyrosine residue in angiotensin II receptor activation. J Biol Chem 270: 9702-9705.

# 63 Han HM, Shimuta SI, Kanashiro CA, Oliveira L, Han SW, et al. Residues Val254, His256, and Phe259 of the angiotensin II AT1 receptor are not involved in ligand binding but participate in signal transduction. Mol Endocrinol 12: 810-814.

# 64 Miura S, Feng YH, Husain A, Karnik SS (1999) Role of aromaticity of agonist switches of angiotensin II in the activationof the AT1 receptor. J Biol Chem 274: 7103-7110.

# 65 Heerding JN, Yee DK, Krichavsky MZ, Fluharty SJ (1998) Mutational analysis of the angiotensin type 2 receptor: contribution of conserved amino acids in the region of the sixth transmembrane domain. Regul Pept 74: 113–119.

# 66 Gribouval O, Gonzales M, Neuhaus T, Aziza J, Bieth E, et al. (2005) Mutations in genes in the renin-angiotensin system are associated with autosomal recessive renal tubular dysgenesis. Nat Genet 37: 964–968.

# 67 Miura S, Zhang J, Boros J, Karnik SS (2003) TM2-TM7 interaction in coupling movement of transmembrane helices to activation of the angiotensin IItype-1 receptor. J Biol Chem 278: 3720-3725.

# 68 Seta K, Sadoshima J (2003) Phosphorylation of tyrosine 319 of the angiotensin II type 1 receptor mediates angiotensin II-induced trans-activation of the epidermal growth factor receptor. J Biol Chem 278: 9019-9026.
